# Integrated analysis of dysregulated microRNA and mRNA expression in intestinal epithelial cells following ethanol intoxication and burn injury

**DOI:** 10.1038/s41598-021-99281-1

**Published:** 2021-10-12

**Authors:** C. J. Herrnreiter, X. Li, M. E. Luck, M. J. Zilliox, Mashkoor A. Choudhry

**Affiliations:** 1 Alcohol Research Program, Loyola University Chicago Health Sciences Division, Maywood, IL 601553 USA; 2 Burn & Shock Trauma Research Institute, Loyola University Chicago Health Sciences Division, Maywood, IL 601553 USA; 3 Department of Surgery, Loyola University Chicago Health Sciences Division, Maywood, IL 601553 USA; 4 Biochemistry and Molecular Biology Program, Loyola University Chicago Health Sciences Division, Maywood, IL 601553 USA; 5 Integrative Cell Biology Program, Loyola University Chicago Health Sciences Division, Maywood, IL 601553 USA; 6 Department of Ophthalmology, Loyola University Chicago Health Sciences Division, Maywood, IL 601553 USA; 7 Department of Microbiology and Immunology, Loyola University Chicago Health Sciences Division, Maywood, IL 601553 USA; 8 Stritch School of Medicine, Loyola University Chicago Health Sciences Division, Maywood, IL 601553 USA; 9 Burn & Shock Trauma Research Institute, Stritch School of Medicine, Loyola University Chicago Health Sciences Division, Bldg. 115/CTRE, Room 320, 2160 South First Ave, Maywood, IL 60153 USA

**Keywords:** Inflammation, Cell signalling, Cell biology, Immunology, Gastroenterology, Pathogenesis

## Abstract

Gut barrier dysfunction is often implicated in pathology following alcohol intoxication and burn injury. MicroRNAs (miRNAs) are negative regulators of gene expression that play a central role in gut homeostasis, although their role after alcohol and burn injury is poorly understood. We performed an integrated analysis of miRNA and RNA sequencing data to identify a network of interactions within small intestinal epithelial cells (IECs) which could promote gut barrier disruption. Mice were gavaged with ~ 2.9 g/kg ethanol and four hours later given a ~ 12.5% TBSA full thickness scald injury. One day later, IECs were harvested and total RNA extracted for RNA-seq and miRNA-seq. RNA sequencing showed 712 differentially expressed genes (DEGs) (padj < 0.05) in IECs following alcohol and burn injury. Furthermore, miRNA sequencing revealed 17 differentially expressed miRNAs (DEMs) (padj < 0.1). Utilizing the miRNet, miRDB and TargetScan databases, we identified both validated and predicted miRNA gene targets. Integration of small RNA sequencing data with mRNA sequencing results identified correlated changes in miRNA and target expression. Upregulated miRNAs were associated with decreased proliferation (miR-98-3p and miR-381-3p) and cellular adhesion (miR-29a-3p, miR-429-3p and miR3535), while downregulated miRNAs were connected to upregulation of apoptosis (Let-7d-5p and miR-130b-5p) and metabolism (miR-674-3p and miR-185-5p). Overall, these findings suggest that alcohol and burn injury significantly alters the mRNA and miRNA expression profile of IECs and reveals numerous miRNA–mRNA interactions that regulate critical pathways for gut barrier function after alcohol and burn injury.

## Introduction

Burn injury is one of the leading causes of accidental injury in the United States, contributing to approximately half a million cases and 40,000 hospitalizations each year^1^. Sepsis and multiple organ failure, resulting from gut barrier disruption and bacterial translocation, are the most prominent cause of death among patients with severe burn injury^2–5^. Alcohol use is a common confounding factor in many traumas, as it increases the risk of accidental injury^6^. Similar to other traumas, nearly half of reported burn injuries occur under the influence of alcohol^7^. Compared to patients with similar burn size and depth, individuals intoxicated at the time of burn injury require longer hospital stays and surgical procedures. Additionally, they have an increased risk of infection, sepsis, multiple organ failure, and ultimately higher mortality rates^6–10^. Therefore, alcohol intoxication at the time of burn injury is not only prevalent, but also significantly contributes to worsened patient outcomes. Consequently, the mechanisms underlying this worsened pathology are an important facet of trauma research that requires further study. To investigate how alcohol exacerbates burn pathophysiology, our laboratory utilizes a well-established mouse model of acute alcohol intoxication and burn injury. Using this model, our laboratory and others have shown that alcohol worsens intestinal inflammation, gut barrier disruption and bacterial dysbiosis after burn injury, which can contribute to the increased risk of sepsis seen in patients^8–10^.

MicroRNAs (miRNAs) are small noncoding RNAs which post-transcriptionally regulate gene expression via complementary binding to the 3′ untranslated region (UTR) of their target mRNAs. This interaction negatively impacts gene expression by either translational repression or mRNA degradation^11,12^. Over half of the genome is estimated to be regulated by miRNAs which regulate key signaling networks in a wide variety of cell types^13,14^. Recent studies demonstrate that miRNAs play a crucial role in regulating intestinal homeostasis and inflammation^15,16^. In addition, dysregulation of numerous miRNAs has been linked to worsened disease in models of chronic intestinal inflammation, including Inflammatory Bowel Disease (IBD) and colorectal cancer^17–19^. Far less is known about the roles of miRNA in models of acute injury and inflammation, such as trauma and burn injury. Moreover, the contributions of miRNA in regulating intestinal barrier dysfunction following alcohol and burn injury are poorly understood. Previous studies in our laboratory show decreased expression of miR-150 in intestinal epithelial cells (IECs) after alcohol and burn injury^20^. Additionally, we see decreased expression of Drosha and Argonaute 2 (Ago2) in intestinal epithelial cells after alcohol and burn injury^20^. Both Drosha and Ago2 are components of the miRNA biogenesis pathway and changes in their expression could have a significant impact on the levels of numerous miRNAs. Furthermore, studies looking at intestinal knockout of Dicer-1, an essential miRNA processing enzyme, show disruption of tight junction protein expression and localization, increased apoptosis, and increased intestinal inflammation^15^. This indicates that global changes in miRNA expression can adversely affect intestinal inflammation and barrier integrity and highlights the need for further research into the impact of miRNA on post-burn intestinal dysfunction.

To assess the global profile of miRNA and begin to evaluate miRNA as a molecular mechanism behind intestinal barrier disruption after alcohol and burn injury, we performed an integrated analysis of miRNA and mRNA expression in intestinal epithelial cells using our well-established mouse model. Small RNA sequencing analysis, also known as miRNA-seq, was used to generate a miRNA expression profile of intestinal epithelial cells one day following alcohol intoxication and burn injury. In addition, we performed mRNA sequencing (RNA-seq) in parallel to assess gene expression. To understand how changes in miRNA expression would impact diverse networks of gene expression, we then integrated miRNA-seq data with the RNA sequencing data to assess correlated changes in miRNA expression and their gene targets. Our findings suggest that IEC miRNA expression is considerably and globally impacted by alcohol and burn injury and is therefore likely to significantly contribute to post-burn pathogenesis. Furthermore, integrated analysis performed using correlated changes in gene and miRNA expression alongside databases of both validated and predicted miRNA gene targets, allowed us to identify mRNA–miRNA interactions relevant to intestinal barrier function, which could play a critical role in perpetuating gut barrier disruption following alcohol exposure and burn injury.

## Results

### Altered miRNA expression in intestinal epithelial cells after ethanol intoxication and burn injury

**Figure 1:**
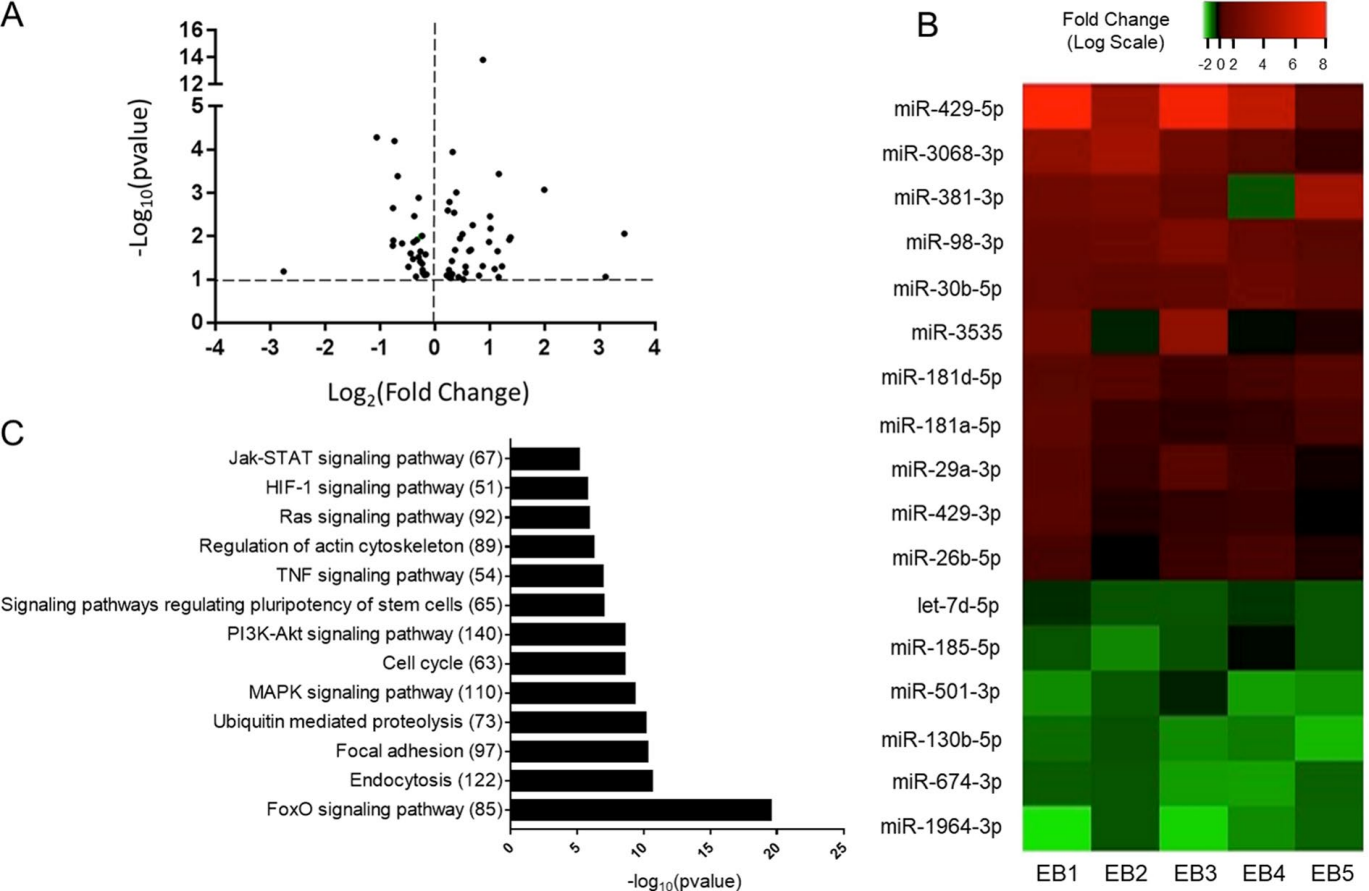
Small RNA sequencing analysis to identify differentially expressed microRNAs in small intestinal epithelial cells 1 day after alcohol and burn injury. (**A**) Volcano plot of differentially expressed microRNAs (p < 0.1) where each point on the graph represents an individual microRNA. Log2 fold change between ethanol burn and sham vehicle treated mice is plotted on the x-axis and the − log10 of the padj value is plotted on the y-axis. Overall, small RNA sequencing analysis identified 65 differentially expressed microRNAs (including 37 upregulated and 28 downregulated). (**B**) Heat map of fold change expression (log scale) of 17 differentially expressed miRNAs (DEMs) in each ethanol burn sample (EB1-5) relative to sham vehicle controls. Each row shows the fold change (log scale) of an individual DEM (padj < 0.1), ranging from downregulated (green) to upregulated (red) (**C**) KEGG pathway analysis of validated microRNA gene targets utilizing miRnet. Each bar represents a KEGG pathway, with the number of associated gene targets in parentheses, found to be significantly enriched (p value < 0.05) among the validated gene targets of the 17 DEMs outlined in (**B**).

**Figure 2:**
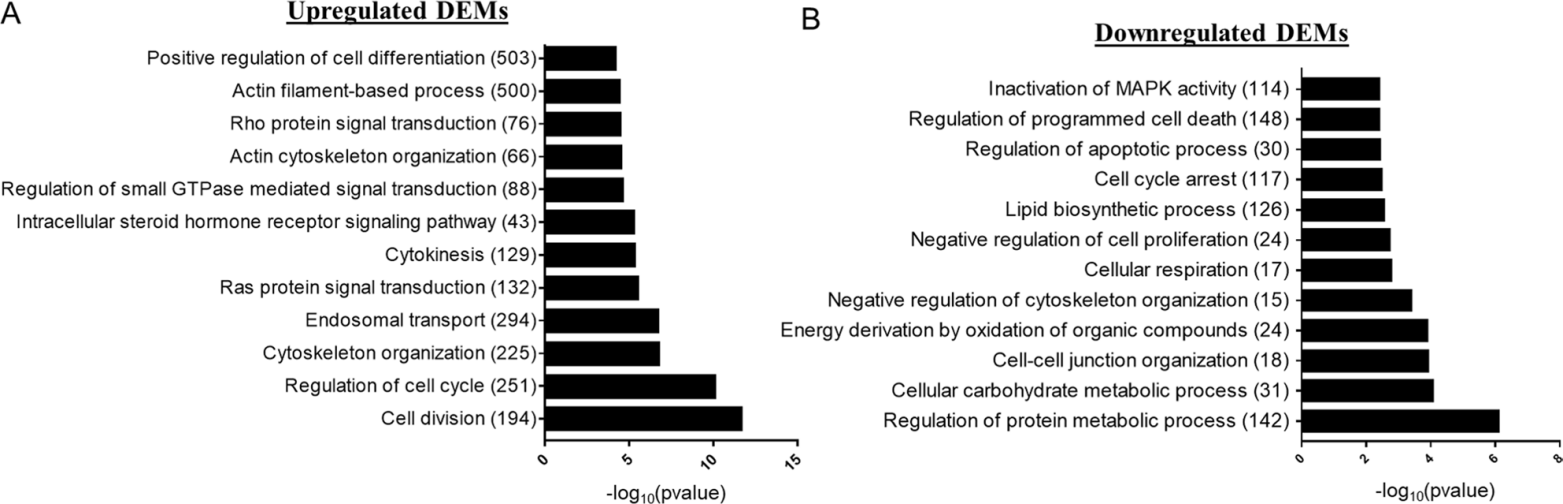
Gene Ontology Enrichment Analysis of Upregulated and Downregulated DEMs. Validated miRNA gene targets for differentially expressed miRNAs were separated into those associated with upregulated DEMs versus downregulated DEMs. GO-BP gene set enrichment analysis was then performed using miRnet built in hypergeometric testing. Significantly enriched (p value < 0.1) pathways of interest are depicted in the bar graphs above for gene targets of either (**A**) significantly upregulated or (**B**) significantly downregulated microRNAs. Numbers in parentheses for each pathway represent to number of identified gene targets associated with the GO-BP pathway term.

To examine the overall impact of ethanol intoxication and burn injury on miRNA expression in the gut, we performed miRNA-seq analysis of small intestinal epithelial cells isolated 24 h after either vehicle treatment and sham injury (n = 5) or ethanol treatment and burn injury (n = 5). Preliminary differential expression analysis (p < 0.1) yielded 65 microRNAs with altered expression in ethanol burn mice compared to sham vehicle (Fig. 1A). To further narrow our results, we adjusted p values using Benjamin–Hochburg to control for multiple testing. Using this method, we identified 17 differentially expressed microRNA (DEMs), 11 of which were significantly upregulated and 6 of which were significantly downregulated (padj < 0.1) (Fig. 1B). To elucidate the potential role of these DEMs in intestinal homeostasis after ethanol and burn injury, we utilized the miRNet Database to assess validated gene targets. The database found at miRNet is a powerful online tool that incorporates data from two widely utilized databases for experimentally validated miRNA gene targets, miRTarBase and TarBase (v8.0)^21,22^. Out of the 17 DEMs, 12 miRNAs (miR-1964-3p, miR-501-3p, Let-7d-5p, miR-185-5p, miR-30b-5p, miR-29a-3p, miR-429-5p, miR181d-5p, miR-429-3p, miR-26b-5p, miR-181a-5p, and miR-381-3p) were present in the miRNet Database. In total, the 5,068 validated gene targets were identified for these 12 DEMs. We then performed functional enrichment analysis on these targets using miRNet’s built in hypergeometric testing with both the KEGG and Gene Ontology (GO) databases. Overall, KEGG pathway analysis showed that our DEMs are associated with several important signal transduction pathways including forkhead box family proteins (Foxo), mitogen-activated protein kinases (MAPK), phosphatidylinositol 3-kinases (PI3K), Akt (also known as protein kinase B or PKB), tumor necrosis factor (TNF), hypoxia-inducible factor-1 (HIF-1), Janus kinases (JAKs) and signal transducer and activator of transcription proteins (STATs). In addition, we found enrichment of genes associated with endocytosis, focal adhesions, regulation of the actin cytoskeleton, ubiquitin mediated proteolysis and the cell cycle (Fig. 1C). To distinguish between pathways that are potentially upregulated or downregulated after alcohol and burn injury, we separated the upregulated DEMs from those that were downregulated. Then, we performed functional enrichment analysis on the validated gene targets identified via miRNet using GO biological process (BP) terms. We found the gene targets specific to upregulated miRNAs were highly associated with proliferation and cell differentiation, as well as the cytoskeleton and actin organization (Fig. 2A). Predicted gene targets for significantly downregulated miRNAs were most associated with metabolism involving proteins, lipids and carbohydrates, cell–cell junction organization, cell cycle arrest and cell death (Fig. 2B).

### Impact of ethanol and burn injury on transcriptional profile of intestinal epithelial cells

**Figure 3:**
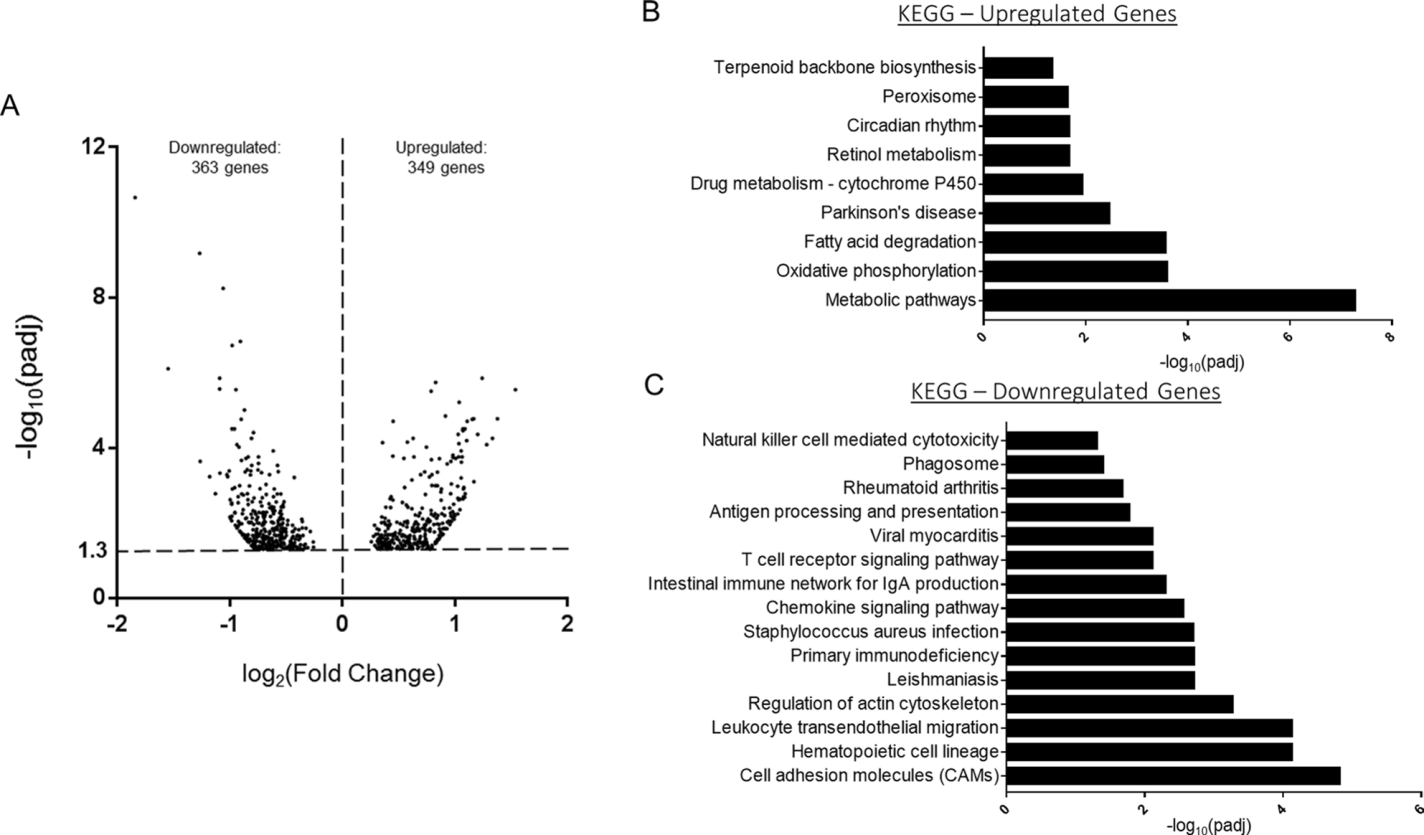
mRNA sequencing analysis of small intestinal epithelial cells 1 day after alcohol and burn injury. (**A**) Volcano plot of differentially expressed genes (padj < 0.05) where each point on the graph represents an individual gene. Log2 fold change between ethanol burn and sham vehicle treated mice is plotted on the x-axis and the − log10 of the padj value is plotted on the y-axis. Overall, mRNA sequencing analysis identified 712 differentially expressed genes (including 349 upregulated genes and 363 downregulated genes). (**B**,**C**) KEGG Pathway enrichment analysis of differentially expressed genes. Each bar represents a KEGG pathway found to be significantly enriched (p < 0.05) in either (**B**) upregulated or (**C**) downregulated genes. All significant pathways (p < 0.05) are shown.

To further delineate the impact of alcohol and burn injury on gene expression, we performed RNA-seq alongside the miRNA sequencing, using the same samples to again compare small intestinal epithelial cells from sham vehicle mice (n = 5) and ethanol burn mice (n = 5). Sequencing achieved an average of 45.7 million cleaned reads per sample and an average mapping rate of 94.9%. In total, we identified 11,809 expressed genes (FPKM > 1), of which 11,078 were expressed by both treatment groups (93.8%). Differential gene expression analysis was then performed using DESeq2 and identified 712 differentially expressed genes (DEGs), including 349 genes significantly upregulated and 363 genes significantly downregulated in ethanol burn mice compared to sham vehicle (padj < 0.05) (Fig. 3A). To assess the functional impact of these gene expression changes and the differences between downregulated and upregulated genes, we utilized KEGG pathway analysis. KEGG pathway analysis of upregulated DEGs demonstrates significant enrichment (padj < 0.05) of metabolic pathways, including oxidative phosphorylation, fatty acid degradation, cytochrome p450 drug metabolism and the peroxisome (Fig. 3B). On the other hand, downregulated DEGs showed significant enrichment (padj < 0.05) in a wide variety of pathways, including cell adhesion molecules, regulation of the actin cytoskeleton, and chemokine signaling (Fig. 3C).

### Analysis of miRNA–mRNA interactions via integration of sequencing data

**Figure 4:**
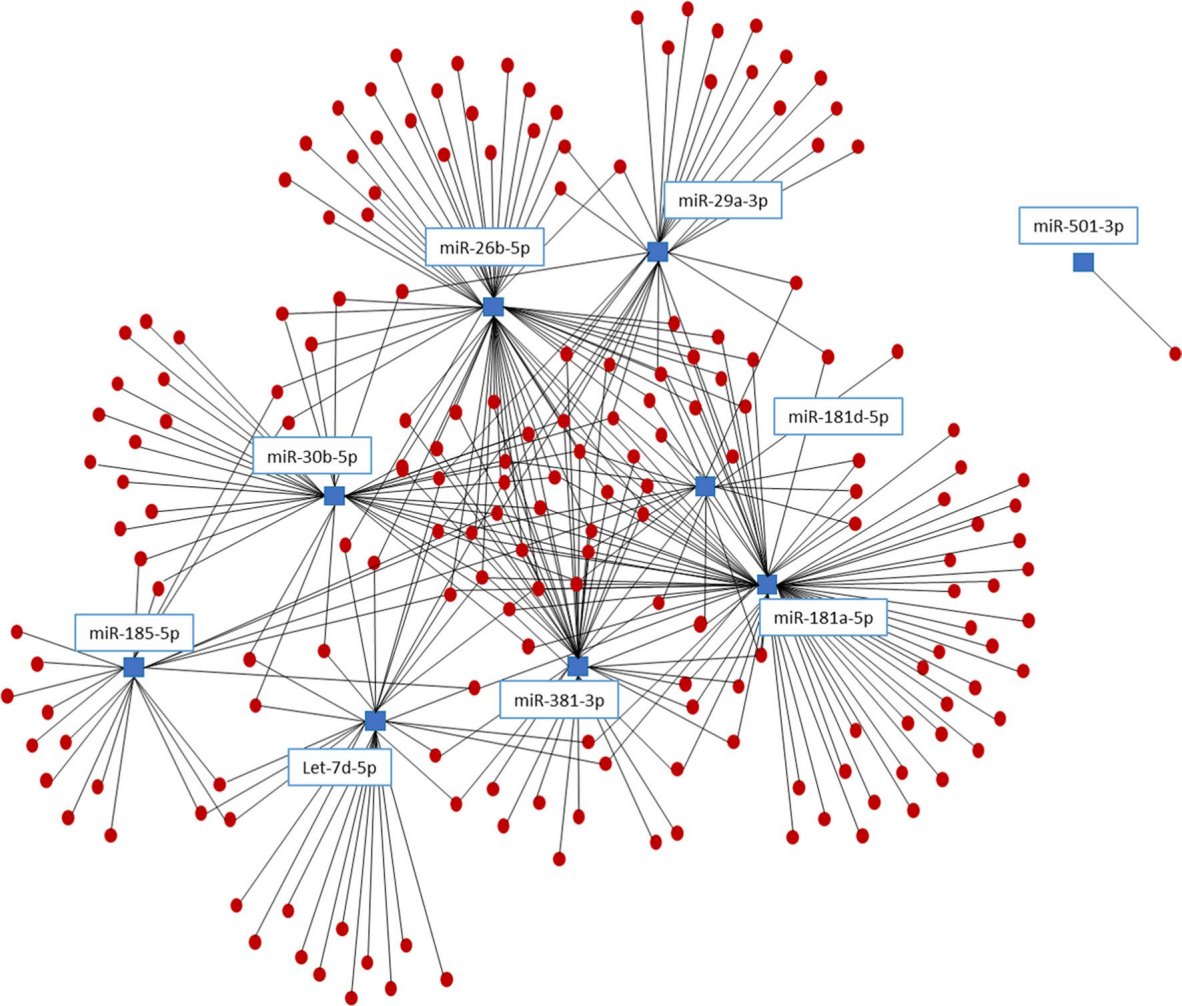
Integrated network of miRNAs and their predicted gene targets which are differentially expressed in small intestinal epithelial cells one day after alcohol and burn injury. miRNA–mRNA interaction network constructed using miRNet containing DEMs (blue squares) and their validated miRNA gene targets which are differentially expressed genes (DEGs) via mRNA sequencing (red circles).

Complex networks of miRNA and their gene targets regulate a wide range of signaling pathways. An individual miRNA can have several different mRNA targets and, therefore, regulate a diverse selection of pathways. In addition, a single mRNA transcript can be targeted by multiple miRNA molecules. Consequently, networks of miRNAs cooperate to significantly impact gene expression and subsequent cellular signaling. To visualize the global range of miRNA and gene target interactions most impacted by alcohol and burn injury, we constructed an interaction network that integrates both our miRNA and RNA sequencing data. To begin, we assessed the 5068 validated gene targets identified previously for our DEMs (Fig. 1) utilizing the miRNet database for overlap with genes identified as differentially expressed by RNA sequencing. Of the 712 DEGs present in our sequencing data, 188 (26.4%) genes were validated targets of 9 of our differentially expressed miRNAs. A miRNA–mRNA network was then generated via miRNet to visualize the interactions between dysregulated genes and miRNAs after alcohol and burn injury (Fig. 4). The miRNet database provides a several measurements of network connectivity and interactions for each gene and miRNA node, including node degree, or the number of nodes directly connected to a particular node, and betweenness centrality, which is a more global look at network structure measuring the number of shortest paths through a node (Supplementary Table ST1). To assess the functional impact of the most connected, core DEMs and their gene targets, we performed GO-BP enrichment analysis on central network genes (degree > 3, betweenness > 100) using g:Profiler with g:SCS multiple testing correction method^23^. Significantly enriched pathways (padj < 0.05) include cell adhesion, proliferation, metabolism, and signaling pathways associated with stress and inflammatory responses (Table 1).

**Table 1:** Integrated miRNA-mRNA network analysis.

GO:BP term	GO:BP ID	padj	Intersections
Cell differentiation	GO:0030154	9.16E−07	ETS1, CHSY1, ZBED6, SPAG9, IKZF1, LIF, LGALS8, DCLK1, ZFP36, EFNB2, MEF2C, IMPACT, JAK2, SEMA6D, CAMSAP2
Cellular metabolic process	GO:0044237	4.44273E−05	TNRC6A, CYB5B, ETS1, CHSY1, ZBED6, SPAG9, IKZF1, MAP3K2, LIF, LGALS8, DCLK1, ZFP36, EFNB2, MEF2C, LCORL, IMPACT, JAK2, DUSP5, PER2
Cell migration	GO:0016477	5.64296E-05	ETS1, SPAG9, LGALS8, DCLK1, EFNB2, MEF2C, JAK2, SEMA6D, ITGAL
Cell development	GO:0048468	0.000220529	CHSY1, SPAG9, LIF, DCLK1, EFNB2, MEF2C, IMPACT, JAK2, SEMA6D, CAMSAP2
Regulation of cell adhesion	GO:0030155	0.002181424	ETS1, LIF, LGALS8, EFNB2, JAK2, ITGAL
MAPK cascade	GO:0000165	0.003589817	SPAG9, MAP3K2, LIF, ZFP36, MEF2C, DUSP5
Cell activation	GO:0001775	0.026985785	IKZF1, LGALS8, EFNB2, MEF2C, JAK2, ITGAL
Regulation of cell population proliferation	GO:0042127	0.035255878	ETS1, LIF, ZFP36, EFNB2, MEF2C, JAK2, ITGAL
Regulation of cell death	GO:0010941	0.035383544	ETS1, ZBED6, ZFP36, EFNB2, MEF2C, IMPACT, JAK2
Cellular response to stress	GO:0033554	0.042570839	TNRC6A, ETS1, SPAG9, ZFP36, MEF2C, IMPACT, JAK2

GO-BP database analysis of central network genes (Degree > 3, Betweenness centrality > 100) was performed using g:Profiler with g:SCS multiple testing correction method. Each term identified in the table are among those significantly enriched (padj > 0.05) with important impacts on intestinal barrier integrity.

**Figure 5:**
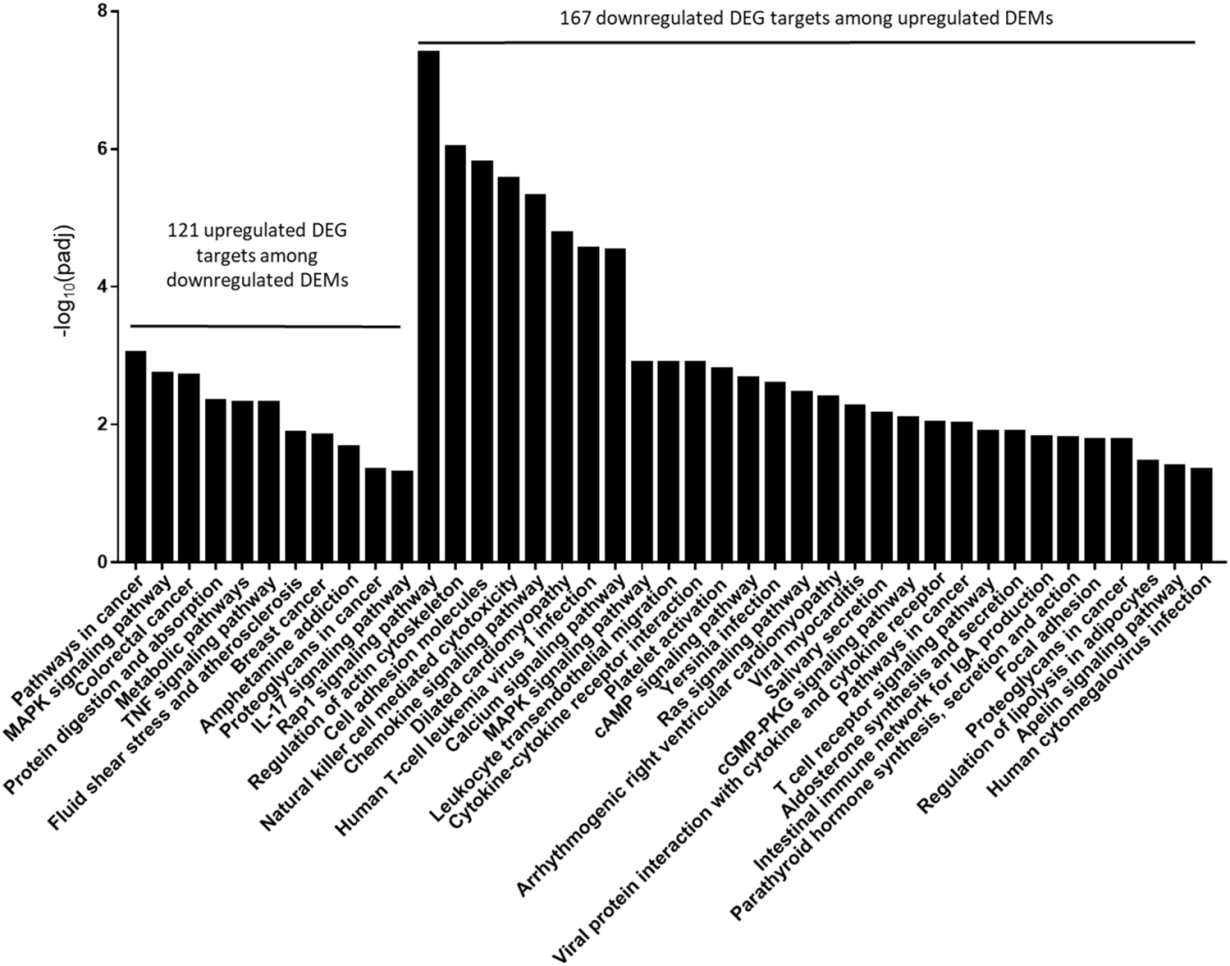
Pathway enrichment analysis using integrated analysis of differentially expressed miRNAs and gene targets with correlated expression changes. Validated and predicted gene targets for each identified DEM were collected from miRNet, TargetScan, and miRDB databases. Gene target lists were then integrated with gene expression data from mRNA sequencing. Gene targets with significantly upregulated expression were extracted for each downregulated DEM, while significantly downregulated gene targets were extracted for each upregulated DEM. The resulting upregulated and downregulated gene targets were compiled separately, and KEGG pathway enrichment analysis was performed using G:profiler with g:SCS multiple testing correction. All significantly enriched (padj < 0.05) pathways are shown in the bar graph above.

As an alternative approach to elucidate potentially important gene targets for individual DEMs after alcohol and burn injury, we performed a separate integrated analysis of each DEM with our RNA sequencing data. As some of our DEMs did not have experimentally validated gene targets in the miRnet database, we expanded our analysis to include predicted gene targets for each DEM from the TargetScan and miRDB databases^24,25^. We then identified which targets exhibited negatively correlated differential expression via RNA sequencing analysis (i.e. for downregulated DEMs we extracted significantly upregulated genes). The results from this data aggregation is shown in Supplementary Tables ST2 and ST3. Biological pathways associated with the identified interactions for each DEM were then assessed by KEGG pathway enrichment analysis via g:Profiler with g:SCS multiple testing correction. To obtain a broad understanding of the regulatory network, we began by combining all identified targets for downregulated versus upregulated DEMs. For upregulated miRNAs, we found a total of 167 predicted gene targets that were downregulated by RNA sequencing. The significantly enriched KEGG pathways for these genes included Rap1 signaling, cellular adhesion, calcium signaling, and hormone signaling, including aldosterone, parathyroid hormone and apelin (Fig. 5). On the other hand, we identified 121 upregulated predicted gene targets for downregulated miRNAs, which were linked to metabolic pathways, TNF signaling, IL-17 signaling, and genes associated with colorectal cancer (Fig. 5). In order to gain a deeper understanding of the role each miRNA could play in gut barrier dysfunction after alcohol and burn injury, we also isolated the predicted gene targets with correlated expression for individual DEMs (Supplementary Tables ST2 and ST3) and again performed pathway enrichment analysis. Supplementary Table ST4 demonstrates that downregulated DEMs miR-674-3p and miR-185-5p exhibited the highest number of significantly associated KEGG pathways. In particular, upregulated gene targets of miR-185-5p were significantly associated with pathways that could disrupt intestinal barrier integrity, including bacterial invasion of epithelial cells and TNF signaling. Other downregulated DEMs exhibited either no significant pathway enrichment among upregulated gene targets, or just one significantly enriched pathway. For example, the peroxisome pathway was the only significantly enriched KEGG term found for Let-7d-5p associated genes. Supplementary Table ST5 demonstrates the plethora of KEGG pathways significantly associated with the downregulated gene targets of our upregulated DEMs. The most common KEGG pathways associated with upregulated DEMs included cell adhesion and tight junctions (miR-29a-3p, miR-429-3p, and miR-3535), Ras signaling (miR-429-3p and miR-26b-5p) and the cell cycle (miR-98-3p and miR-381-3p). In addition, we saw significant enrichment in hormone signaling pathways associated with GI motility and mucosal function, including oxytocin (miR-181a-5p, miR-98-3p and miR-381-3p), aldosterone (miR-429-5p, miR-181a-5p and miR-98-3p), and parathyroid hormone (miR-98-3p and miR-381-3p).

## Discussion

Disruption of the gut barrier after alcohol intoxication and burn injury is known to contribute to worsened patient outcomes, including sepsis, multiple organ failure and ultimately death^2–8^. Although previous studies demonstrate reduced expression of key miRNA processing enzymes Drosha and Ago2 in intestinal epithelial cells after alcohol and burn injury, the influence of miRNA dysregulation on gut barrier function following acute trauma is understudied. The goal of this report was to assess the global impact of alcohol and burn injury on intestinal epithelial cell miRNA expression and gain deeper insight into the potential mechanisms by which miRNA could contribute to gut barrier disruption following combined injury. We found widespread changes in miRNAs and their target gene expression within intestinal epithelial cells after alcohol and burn injury. Moreover, integration of miRNA and mRNA sequencing data revealed several interactions which are highly associated with intestinal barrier integrity and provide evidence that changes in miRNA expression following alcohol intoxication and burn injury are an important component of post-burn pathogenesis.

Intestinal epithelial cells form a crucial physical barrier, which maintains homeostasis between pathogens present in the microbiome and the host defense response^3,26^. Disruption of this barrier promotes severe complications after burn injury, including sepsis and multiple organ failure^2,4^. Following alcohol and burn injury, intestinal barrier disruption is primarily instigated by heightened inflammation, loss of tight junction proteins, reduced IEC proliferation and increased IEC apoptosis^27,28^. In line with the literature, our RNA sequencing data shows significant association between downregulated genes and cellular adhesion pathways (Fig. 3C). Under normal conditions, intestinal homeostasis is maintained, in part, through complex interactions between miRNAs and their gene targets, which coordinate signaling pathways critical for the regulation of IEC apoptotic and inflammatory signaling, in addition to the preservation of tight junction stability^15^. Our findings show that widespread changes in miRNA expression after alcohol intoxication and burn injury likely contribute to disruption of normal gut homeostasis. Utilizing miRNet, a database of experimentally validated miRNA gene targets, we found that several of our differentially expressed miRNAs regulate gene targets associated with intestinal barrier integrity (Fig. 1C). Specifically, pathways associated with cellular adhesion, including organization of the actin cytoskeleton and Rho-GTPase signaling, were enriched among predicted gene targets of our upregulated DEMs (Fig. 2A). Additionally, predicted gene targets associated with negative regulation of cytoskeleton organization are enriched among downregulated DEMs (Fig. 2B). While cell adhesion is critical for maintaining an intact intestinal barrier, intestinal epithelial cells are constantly being sloughed off and replaced. This process makes the balance between cellular proliferation and cell death very important. We found that downregulated DEMs were significantly associated with cell cycle arrest and programmed cell death, including apoptosis (Fig. 2B), while cellular division and the cell cycle were among the enriched pathways for the gene targets of upregulated DEMs (Fig. 2A). As miRNAs inhibit the translation of their mRNA targets, we anticipated that these identified changes to the miRNA expression profile of IECs after alcohol and burn injury would consequently promote cell death while reducing proliferation and cellular adhesion. To further elucidate which of these DEMs are most likely contributing to intestinal dysfunction after alcohol and burn injury, we utilized two separate methods to assess correlated changes in the expression of DEMs and their gene targets. From this, we identified numerous interactions related to regulation of gut barrier integrity, including interactions which would reduce cellular proliferation (increased miR-98-3p and miR-381-3p), and adhesion (increased miR-29a-3p, miR-429-3p and miR-3535) while increasing inflammation (reduced miR-130b-5p, miR-674-3p, miR-185-5p and Let-7d-5p) and cell death (Let-7d-5p and miR-130b-5p) (Table 1, Fig. 5, and Supplementary Tables ST4 and ST5).

Following the acute phase of injury, burn patients must content with systemic and chronic hypermetabolic responses for up to years after injury. This hypermetabolic state is characterized by increased energy expenditure and metabolic rates, full body catabolism, muscle protein degradation, elevated adipose lipolysis, and insulin resistance^29–31^. Studies suggest this process is mediated by increases in both plasma catecholamine and corticosteroid levels and elevated system pro-inflammatory mediators^29,32,33^. It is also known that increased inflammation following chronic alcohol consumption negatively impacts insulin signaling, thereby also contributing to increases in metabolic processes^33,34^. Although both burn injury and chronic alcohol consumption promote hypermetabolism, there are few studies which explore the impact of combined alcohol intoxication and burn injury on metabolic outcomes. One recent study revealed significant alterations in metabolism after combined insult, including hyperglycemia and changes in serum insulin, glucagon and gastrointestinal hormones in alcohol and burn mice compared to sham or burn alone^35^. In our study, almost all KEGG pathways significantly associated with upregulated genes in IECs after alcohol and burn injury were metabolic in nature (Fig. 3B). The gastrointestinal tract is the initial sight of nutrient absorption and metabolism in the body. Proper metabolic function of intestinal epithelial cells is critical for both gut homeostasis and barrier function. For example, glucagon-like peptide-2 (GLP-2) is a hormone produced in the gastrointestinal epithelium that is important for nutrient absorption, lipid metabolism and energy homeostasis. However, studies also show GLP-2 promotes intestinal barrier integrity by stimulating IEC proliferation, inhibiting apoptosis, and increasing tight junction protein expression^36,37^. In addition to the levels of a variety of nutrients, lipid and bile acid metabolism have also been shown to impact gut barrier function and inflammation^38–41^. Taken together, these findings suggest that intestinal hypermetabolism after alcohol and burn injury likely plays a major role in gut dysfunction. In the last decade, miRNAs have emerged as crucial regulators of metabolism in the liver, pancreas, muscle, and adipose tissue. Dysregulation of miRNA expression has been linked to several metabolic disorders, including obesity and diabetes^42,43^. More recent studies demonstrate a clear relationship between intestinal epithelial cell metabolism and miRNAs. Small intestinal miRNA expression is significantly altered by high fat diet and excessive lipid exposure^43,44^. Furthermore, a study utilizing a mouse model of intestinal Dicer-1 knockout showed that disruption of intestinal miRNA expression results in changes to lipid absorption and accumulation^45^. Other studies have confirmed extensive regulation of lipid metabolism by intestinal miRNAs^46^. Although research exploring miRNA mediated regulation of intestinal metabolism in the context of alcohol and burn injury is lacking, our findings highlight intestinal epithelial cell metabolism as an important network impacted by miRNA changes after alcohol and burn injury. In accordance with the repressive nature of miRNAs, we found metabolic pathways (lipid biosynthesis, cellular respiration, energy derivation by oxidation of organic compounds, carbohydrate and protein metabolism) enriched among the miRNet derived validated gene targets of our downregulated DEMs (Fig. 2B). When we integrate gene expression data from RNA sequencing and extract the validated gene targets which also exhibit differential expression, the resulting network also exhibited significant association with regulation of metabolic processes (Table 1). This association with metabolism was further demonstrated in a separate integrated analysis which integrated both validated and predicted gene targets of DEMs with RNA sequencing data (Fig. 5, Supplementary Table ST2–ST5). Individual assessment of each DEM identified miR-674-3p and miR-185-5p as critical downregulated miRNAs with upregulated gene targets associated with lipid and protein metabolism (Supplementary Table ST4). Future studies investigating the mechanism by which these miRNAs influence IEC metabolism after alcohol and burn injury could provide new insights into their impact on gut barrier integrity.

A limitation of this study is the lack of ethanol alone or burn injury alone groups for comparison. However, several previous studies by our laboratory have established that alcohol or burn injury (~ 12.5%) alone do not have any significant effect on intestinal pathophysiology and show no changes in barrier function^47–49^. Furthermore, a recent study demonstrates that gene expression of microRNA biogenesis components Drosha and Ago2 are significantly downregulated in intestinal epithelial cells after combined insult but not alcohol or burn injury alone^20^. Although the contribution of burn and alcohol insult alone remains to be established, we feel that the findings included in this manuscript provide an adequate overview of global changes in miRNA expression and lay the foundation for future mechanistic studies underlying gut barrier dysfunction following alcohol and burn injury. We further recognize that this study is limited to male mice only and whether gender has any role in altered miRNA expression following alcohol and burn injury remains to be studied.

**Figure 6:**
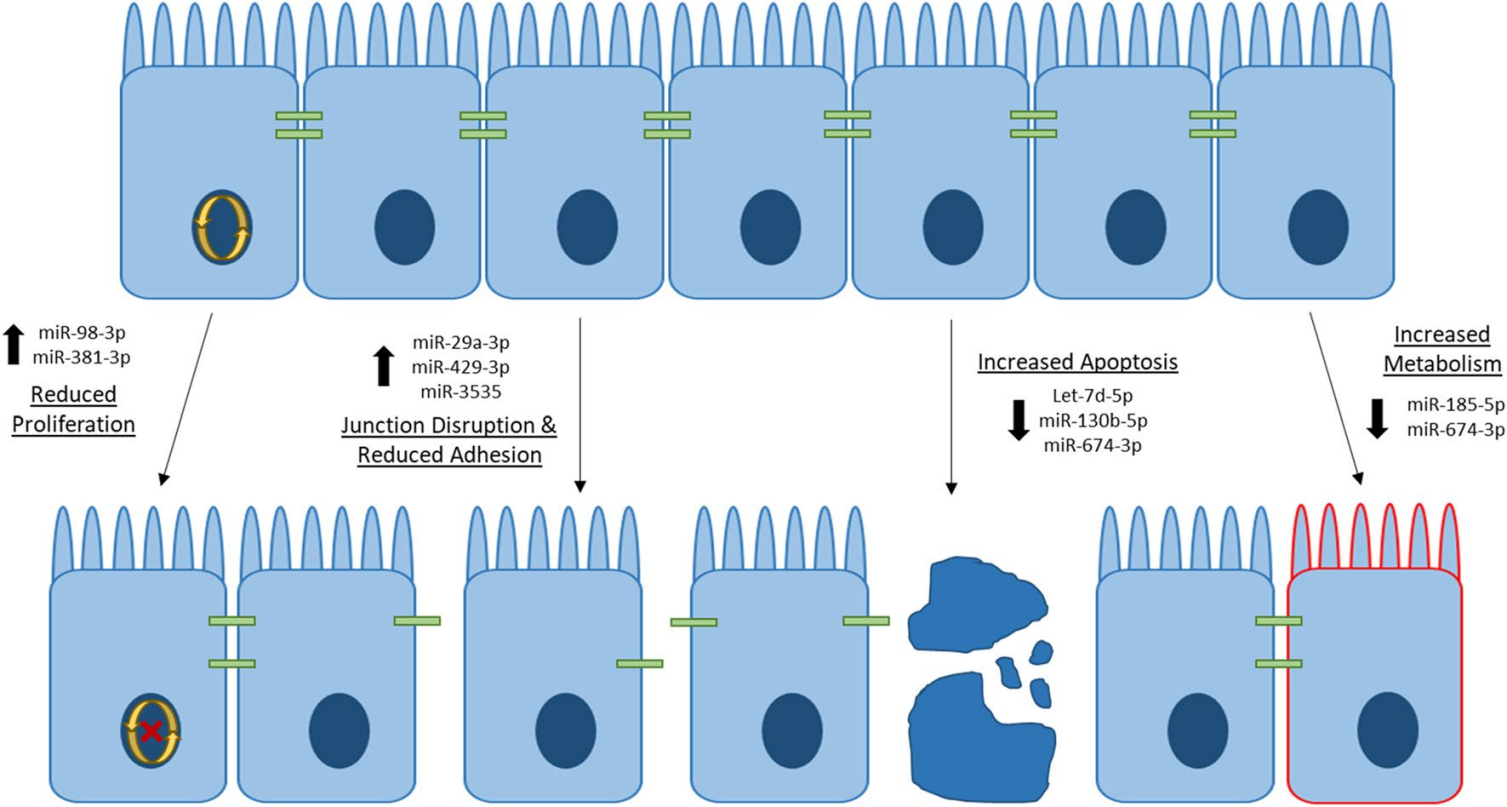
Dysregulated miRNA expression promotes intestinal barrier disruption after alcohol and burn injury. Intestinal epithelial cells maintain the gut barrier via a variety of mechanisms that include the expression of tight junction proteins, strict regulation of cellular metabolism, and balancing cell death with cellular proliferation. Changes in these crucial functions results in disruption of the gut barrier following alcohol intoxication and burn injury, contributing to sepsis and multiple organ dysfunction. Our study indicates that dysregulated miRNA expression could significantly impact several pathways associated with intestinal barrier function after alcohol and burn injury. Here we particularly highlight upregulated miRNAs that were associated with reduced proliferation (miR-98-3p and miR-381-3p) and tight junctions (miR-29a-3p, miR-429-3p and miR3535), and downregulated miRNAs that were connected to the upregulation of apoptotic (Let-7d-5p and miR-130b-5p) and metabolic signaling (miR-674-3p and miR-185-5p).

Altogether, our results demonstrate significant intestinal miRNA dysregulation following alcohol and burn injury. Furthermore, we revealed several miRNAs of interest, summarized in Fig. 6, which likely play an important role in decreasing gut barrier integrity by reducing intestinal proliferation (miR-98-3p and miR-381-3p), disrupting epithelial cell tight junctions (miR-29a-3p, miR-429-3p and miR-3535), and promoting intestinal apoptosis (Let-7d-5p and miR-130b-5p) and hypermetabolism (miR-674-3p and miR-185-5p). Overall, these finding open the door to future mechanistic studies that could increase our understanding of how miRNAs regulate and control gut barrier function and intestinal homeostasis.

## Methods

### Animals

10–12-week-old C57BL/6 male mice (23–25 g body weight) were obtained from Charles River Laboratories and maintained in animal housing facilities at Loyola University Chicago Health Sciences Division, Maywood, Illinois, USA.

### Murine model of acute alcohol intoxication and burn injury

12 week old male C57/BL6 mice (22–26 g) were randomly assigned into two experimental groups: Sham injury + vehicle (water) (n = 6) or burn injury + ethanol (n = 7). On the day of injury, mice were gavaged with 400 µL of 25% ethanol in water (2.9 g/kg). Sham + vehicle animals were gavaged with 400 µL water. Four hours following the gavage, mice were given 1 mg/kg buprenorphine subcutaneously. Mice were anesthetized with a ketamine hydrochloride/xylazine cocktail (~ 80 mg/kg and ~ 1.2 mg/kg respectively) given by intraperitoneal injection. The dorsal surface of the mice was shaved before placing the mice in a prefabricated template exposing ~ 12.5% total body surface area calculated using Meeh’s formula as described by Walker and Mason(26). Burn group animals were immersed in ~ 85 °C water bath for ~ 7 s to induce a full-thickness scald burn injury. Sham animals were placed in a 37 °C water bath for 7 s. Following burn or sham injury, animals were dried gently and given 1.0 mL normal saline resuscitation by intraperitoneal injection. Animals were returned to their cages and allowed food and water ad libitum. All animal experiments were conducted in accordance with the guidelines set forth in the Animal Welfare Act and ARRIVE. In addition, they were approved by the Institutional Animal Care and Use Committee (IACUC) at Loyola University Health Sciences Division.

### Small intestinal epithelial cell total RNA isolation

One day after injury, mice were euthanized, and the abdominal cavity was exposed via midline incision. ~ 8 cm of the distal small intestine was harvested and opened longitudinally and washed in cold PBS. Small intestines were then incubated in HBSS supplemented with 10 mMol/L HEPES, 50 μg/mL gentamicin, 100 U/mL penicillin, 100 μg/mL streptomycin, 5 mM EDTA and 1 mM DTT (pre-digestion solution) for 20 min at 37 °C with agitation and then vortexed to disrupt epithelial cells from the lamina propria. Epithelial cells were collected through a 100 µm strainer and the disruption process was repeated a second time^50,51^. Isolated epithelial cells were washed and then total RNA extracted using *mir*Vana miRNA Isolation Kit (Invitrogen) according to manufacturer’s instructions. Total RNA concentration and purity were assessed using a Nanodrop spectrophotometer (ThermoScientific).

### miRNA sequencing analysis

Isolated total RNA from small intestinal epithelial cells was submitted to Northwestern University Chicago’s NUSeq Core Facility for library preparation and miRNA sequencing (low quality RNA samples excluded and n = 5 per group chosen randomly). Analysis, including read clean up, genome mapping, and differential expression analysis, were performed at Loyola University by Dr. Michael Zilliox. Briefly, small RNA libraries were prepared using TruSeq Small RNA Library Preparation Kit according to manufacturer’s instructions. Resulting libraries were then sequenced at 100 million raw reads per sample using 75 base pair, single read sequencing via the NextSeq Illumina Platform. Raw sequencing reads were then cleaned using Cutadapt software to remove adaptor sequences and low quality reads^52^. The resulting clean reads were then mapped to the mouse genome, mm10. An annotation file from miRBase release 22.1^53,54^, describing miRNA coordinates, and the sequencing alignment mappings were used as input for the Python package HTSeq to generate raw counts of miRNAs observed in the alignments^55^. Differential expression analysis was performed using the DESeq2 R package and Wald tests were conducted to determine significance^56^. Differentially expressed miRNAs were then identified using p-values both before and after adjustment using Benjamini and Hochberg’s approach to control for the false discovery rate (p value < 0.1 and padj < 0.1). Gene set enrichment analysis was performed using validated miRNA gene targets from the miRNet database^21,22^ and their built in software for Gene Ontology (GO) and KEGG pathway enrichment analysis^57–59^.

### RNA sequencing analysis

Isolated total RNA from small intestinal epithelial cells was submitted to Novogene for library preparation and RNA sequencing analysis (low quality RNA samples excluded and n = 5 per group chosen randomly). Briefly, samples were enriched for mRNAs using oligo(dT) beads targeting the 3′ polyA tail. Enriched mRNAs were then fragmented randomly and non-stranded, non-directional library preparation performed using NEBNext kit according to manufacturer’s instructions. Resulting libraries were sequenced at 20 million raw reads per sample using 150 base pair, pair-ended sequencing via the Illumina Platform. Raw reads were obtained by CASAVA base recognition (Base Calling) and then filtered for adaptor contamination and low quality score. The resulting clean reads were then mapped to the mouse genome using STAR software^60^. FPKM (expected number of Fragments Per Kilobase of transcript sequence per Million base pairs sequenced) was used to quantify mRNA expression and then differential expression analysis was performed using the DESeq2 R package^56^. Differentially expressed genes were identified using p-values adjusted using Benjamini and Hochberg’s approach to control for the false discovery rate (padj < 0.05). Gene set enrichment analysis was performed using clusterProfiler software and the KEGG database^57,58,61^.

## Supplementary Information

Supplementary Information.
