# Supplementary material for: Integrated analysis of dysregulated microRNA and mRNA expression in intestinal epithelial cells following ethanol intoxication and burn injury
Source: Sci Rep. 2021 Oct 12;11:20213. doi: 10.1038/s41598-021-99281-1 (PMC8510995; doi:10.1038/s41598-021-99281-1)
Supplement: Supplementary file 1 — Supplementary Information. [file 41598_2021_99281_MOESM1_ESM.pdf]

Supplemental Tables

	Degree	Betweenness
mmu-mir-181a-5p	80	8370.622
mmu-mir-26b-5p	62	5910.213
mmu-mir-381-3p	41	2739.336
mmu-mir-30b-5p	37	3348.676
mmu-mir-29a-3p	32	3079.026
mmu-let-7d-5p	31	2956.921
mmu-mir-185-5p	22	2265.051
mmu-mir-181d-5p	17	494.1545
Ubn2	6	724.7007
Tnrc6a	6	622.2108
Cyb5b	5	502.6398
Ets1	5	814.2055
Chsy1	5	546.8513
Zbed6	5	639.4428
Spag9	4	164.1109
Zzef1	4	580.5738
Ikzf1	4	287.9076
Map3k2	4	149.2499
Lif	4	280.8466
Gpatch8	4	164.1109
Lgals8	4	392.6627
Marcks	4	265.0895
Dclk1	3	113.8317
Zfp36	3	313.4268
Efnb2	3	168.8103
Mef2c	3	113.8317
Lcorl	3	113.8317
Slc36a1	3	203.1711
Rad21	3	45.96643
Impact	3	105.2462
Jak2	3	138.8257
Sema6d	3	181.5699
Mfsd1	3	113.8317
Luc7l2	3	45.96643
Itgal	3	290.1322
Dusp5	3	320.3269
Snn	3	228.8594
Camsap2	3	181.5699
Fam160a2	3	45.96643
Per2	3	129.5313
Tgoln1	3	92.60234
Runx3	2	99.25461
Ikzf2	2	11.20734
Paxbp1	2	25.92271

	Degree	Betweenness
Fcho2	2	129.1399
Sema5a	2	27.14549
Reep4	2	48.87201
Trak1	2	60.76353
Cyp51	2	70.43002
Dusp3	2	43.23596
Prkacb	2	25.92271
H13	2	67.75013
Fyn	2	77.23449
Lpin1	2	55.0445
Fos	2	11.20734
Lcp1	2	85.85811
Itm2b	2	27.14549
Gtse1	2	48.87201
Cpn2	2	100.2288
Lmnb1	2	69.66699
P4hb	2	11.20734
Pgm2	2	55.0445
Dst	2	100.2288
Fn1	2	51.3062
Cfh	2	60.76353
Slc20a1	2	77.23449
Ptpn22	2	60.76353
Mfsd2a	2	20.14842
Per3	2	60.76353
Ccnl2	2	27.14549
Pik3c2a	2	25.92271
Magt1	2	129.1399
Msmo1	2	71.93436
Sesn3	2	48.87201
Gramd1b	2	85.85811
Zfp704	2	67.75013
Cd53	2	98.42883
Dmxl2	2	77.23449
AW549877	2	69.66699
Myof	2	25.92271
Pom121	2	60.76353
Retsat	2	51.3062
Exoc5	2	60.76353
Cttnbp2nl	2	60.76353
Il2rb	2	69.66699
Ccnd1	2	51.3062
Zfp827	2	27.14549
Gm12258	2	27.14549

Supplemental Table ST1. Connectivity values for DEMs and DEGs present in the integrated network generated via miRNet. Degree and Betweenness measurements calculated by the miRNet software. All nodes with Degree values of 1 exhibit no Betweenness value, and were therefore excluded from this table.

DEM	miRnet	Targetscan	miRDB	All	DEGs
miR-1964-3p	1	544	23	548	Cep85, Clcn6, Cyb5b, Dclk1, Fosb, Micalcl, Ppp1r12b, Tbc1d24
miR-130b-5p	0	4172	724	4355	Aldh1a1, Atxn7, Avil, Bcl2l15, Ccdc134, Ccdc88c, Clcn6, Col11a2, Cyb5b, Cyp3a44, Dclk1, Dlg3, Dst, Efnb2, Exoc5, Fcho2, Fmo2, Fos, Gadd45a, Gramd1b, Gramd2, H13, Hip1r, Hmx2, Impact, Iqgap2, Kctd12, Lcorl, Luc7l2, Mafk, Magt1, Map3k2, Mapk13, Micalcl, Mprip, Myo1a, P4hb, Pfkfb2, Pgm2, Plat, Pmaip1, Ppp1r12b, Ptrf, Ralgps1, Rbp2, Sec16b, Sema6d, Sh2d7, Slc35b1, Slc5a9, Slc6a14, Spag9, Steap4, Tcf7l2, Tef, Tex12, Tmc5, Tnrc6a, Ubn2, Zfp36, Zfp827, Zzef1
miR-501-3p	28	24	234	269	Dclk1,Fos,Mroh6
Let-7d-5p	673	651	804	1587	Acox2, Arhgap12, AW549877, Cpn2, Dmxl2, Dst, Dusp5, Edn1, Fcho2, Fn1, Gramd1b, H13, Hip1r, Homer2, Isoc2a, Lcorl, Lgals8, Magt1, Map3k2, Micalcl, Mroh6, Mvk, Myo7a, Pmaip1, Ppp1r12b, Prpf38b, Ralgps1, Retsat, Rgn, Rorc, Sec16b, Slc20a1, Slc5a9, Stard13, Vwce, Xylt2, Zbed6, Zfp36, Zzef1
miR-674-3p	0	3447	486	3563	Aldh1a1, Alox5ap, Atp1a1, Avil, Camk2b, Ccdc62, Chka, Clca1, Cyb5b, Dlg3, Dnhd1, Dpp4, Dst, Efnb2, Exoc5, Fam213a, Fgf9, Fn1, Gramd2, H13, Hkdc1, Hoga1, Homer2, Impact, Kctd12, Lrrc19, Luc7l2, Map3k2, Per2, Pmaip1, Ppa1, Ppp1r12b, Ptp4a1, Ptrf, Ralgps1, Sec16b, Slc35c2, Slc5a9, Slc6a4, Spag9, St3gal4, Stard13, Ubn2
miR-185-5p	410	229	987	1453	Aldh1a1, Als2cl, Cgn, Cyb5b, Cyp2d9, Cyp4b1, Dnm1, Dpp4, Dusp5, Edn1, Fmo2, Fn1, Lif, Me3, Mettl7b, Pgm2, Retsat, Slc35c2, Slc36a1, Syna, Sypl2, Tmem176a, Ubn2, Xylt2, Zzef1

Supplemental Table ST2. Predicted and validated upregulated gene targets for individual downregulated DEMs. Table shows the number of hits for each downregulated miRNA in both validated (miRNet) and predicted (Targetscan and miRDB) miRNA gene target databases. The "All" column indicates the total number of unique gene target hits when all three databases are aggregated. The final "DEG" column lists the gene targets from all three databases that were shown to be upregulated via RNA sequencing analysis.

DEM	miRnet	Targetscan	miRDB	All	DEGs
miR-30b-5p	929	392	1327	1987	Adrb1, Akna, B2m, Camsap2, Cd8a, Chsy1, Cotl1, Ets1, Fyn, Galnt7, Gtse1, Gzmb, Ikzf1, Ikzf2, Kctd7, Lax1, Lcp2, Lpin1, Marcks, Mcf2l, Plk1, Rab15, Rasgef1b, Reep4, Rgs2, Sesn3, Snx33, Tnfrsf19
miR-29a-3p	953	767	862	1882	9530068E07Rik, Acsf2, Actn2, Antxr2, Atp2b4, Bbs7, Ccl9, Ccr9, Cd53, Chsy1,Cps1, Cst7, Dock11, Dusp18, Dusp3, Emp2, Enpp3, Ets1, Fyn, Igsf9b, Ikzf1, Il2rb, Itgb7, Klhl25, Lcp1, Lmnb1, Mmp15, Mpzl1, Myo1g, Orai2, P2rx4, Pmp22, Pvrl1, Selplg, Sh2d2a, Sidt1, Snn, Srgap2, Tmem229b, Tnfrsf9, Zfp704
miR-3068-3p	0	2383	390	2530	Adcy7, Antxr2, Bace2, Camsap2, Cps1, Cttnbp2nl, Cyp39a1, Dock2, Dusp18, Ect2, Emp2, Endod1, Fcrla, Galnt7, Gpr20, Id4, Igf2, Ikzf1, Ikzf2, Lcp1, Nckap1l, Prelid2, Rab15, Rasgrp3, Rgs2, Rttn, Selm, Selplg, Sema5a, Sesn3, Sidt1, Snx33, Tmem229b, Was, Yars2, Zdhhc2
miR-429-5p	3	2281	266	2351	Adora2a, Atp2b4, Camsap2, Ccr9, Cd177, Cd8a, Cd96, Cps1, Cpt1a, Dcps, Dmrt3, Emp2, Galnt5, Gzmk, Ikzf1, Ikzf2, Lmnb1, Lrrc8c, Mansc1, Mcf2l, Mef2c, Msn, Poc1a, Prkacb, Prkd3, Rab15, Rad21, Sema5a, Sesn3, Spn, Srbd1, Swap70, Tnfrsf13c, Tnfrsf9, Trp53i11, Zdhhc18, Zdhhc2
miR-181d-5p	519	195	1119	1521	Camsap2, Cbfa2t3, Cttnbp2nl, Ets1, Galnt7, Igsf9b, Ikzf1, Ikzf2, Ilf3, Itga1, Mfsd1, Rad21, Rassf2, Snn, Spats2l, Srgn, Tgoln1, Tmem123, Wls, Zfp704
miR-181a-5p	2200	621	1120	2897	Adcy7, Antxr2, Atp2b4, Camsap2, Car2, Cbfa2t3, Cd53, Chsy1, Cttnbp2nl, Ets1, Galnt7, Hmgb2, Igsf9b, Ikzf1, Ikzf2, Ilf3, Itga1, Itgal, Itm2b, Lpcat1, Lsp1, Marcks, Mef2c, Mfsd1, Parm1, Pom121, Prkd3, Rad21, Rassf2, Rgs2, Sema5a, Sesn3, Slc35e4, Snn, Spats2l, Srgap2, Swap70, Tgoln1, Tmem123, Tnfrsf19, Wls, Zfp704
miR-429-3p	34	58	1010	1049	AI467606, Anln, Camsap2, Cep192, Ckap4, Ets1, Fyn, Ikzf2, Lpin1, Marcks, Msn, Myof, P2rx4, Prkacb
miR-26b-5p	1688	183	920	2168	Camsap2, Ccr9, Celsr1, Chsy1, Ckap4, Cks2, Cpt1a, Cttnbp2nl, Dmrt3, Dusp18, Fam46c, Galnt7, Gtse1, Hmgb2, Il2rb, Itga5, Itgal, Kpna2, Lgals1, Lmnb1, Lpin1, Marcks, Mef2c, Mfsd1, Mras, Myof, Pom121, Prkacb, Rac2, Reep4, Runx3, Sesn3, Snn, Spats2l, Tgoln1, Zdhhc18
miR-98-3p	0	892	1383	1951	Bbs7, Camsap2, Cd244, Cdc42ep3, Cep192, Cttnbp2nl, Dock11, Ect2, Emp2, Hic1, Hmgb2, Id4, Ikzf2, Itm2b, Marcks, Mef2c, Mfsd1, Plk1, Prelid2, Prkacb, Prkd3, Rad21, Zfp704
miR-3535	0	1709	240	1791	Anln, Antxr2, Apbb1ip, Atp2b4, Cbfa2t3, Ccr5, Cd52, Cd53, Clca4, Cldn2, Cps1, Cst7, Cttnbp2nl, Fam46c, Gpr20, Gpr55, Grap2, H1f0, H2afz, Ikzf1, Ikzf2, Ikzf3, Itga1, Itgb2, Lphn2, Mef2c, Nckap1l, Nod1, P2rx7, Paip2b, Plp2, Psmd9, Pvrl1, Rad18, Rasgrp3, Rcsd1, Rnase4, Rnf26, Sema5a, Sesn3, Spats2l, Spn, Tlr1, Tmem138, Tnfrsf19
miR-381-3p	1350	237	1070	2100	Asf1b, Cdc42ep3, Cep192, Chsy1, Dock11, Dusp3, Emp2, Ets1, Hmgb2, Ikzf2, Itm2b, Marcks, Mef2c, Mfsd1, Myof, Paip2b, Plk1, Pmp22, Prkacb, Pvrl1, Rad21, Sema5a, Zdhhc2, Zfp704

Supplemental Table ST3. Predicted and validated downregulated gene targets for individual upregulated DEMs. Table shows the number of hits for each upregulated miRNA in both validated (miRNet) and predicted (Targetscan and miRDB) miRNA gene target databases. The “All” column indicates the total number of unique gene target hits when all three databases are aggregated. The final “DEG” column lists the gene targets from all three databases that were shown to be downregulated via RNA sequencing analysis.

Downregulated DEMs	KEGG Pathways Associated with Upregulated DEG Targets	KEGG ID	padj
miR-1964-3p	none	none	none
miR-130b-5p	none	none	none
miR-501-3p	Amphetamine addiction	KEGG:05031	0.096497347
Let-7d-5p	Peroxisome	KEGG:04146	0.075586996
miR-674-3p	Proteoglycans in cancer	KEGG:05205	0.021573313
	Carbohydrate digestion and absorption	KEGG:04973	0.028740452
	Regulation of actin cytoskeleton	KEGG:04810	0.029003959
	Pathways in cancer	KEGG:05200	0.032492754
	Gastric acid secretion	KEGG:04971	0.070900992
	Insulin secretion	KEGG:04911	0.088949703
miR-185-5p	Bacterial invasion of epithelial cells	KEGG:05100	0.027533265
	Retinol metabolism	KEGG:00830	0.043145139
	AGE-RAGE signaling pathway in diabetic complications	KEGG:04933	0.04781807
	Protein digestion and absorption	KEGG:04974	0.055779689
	TNF signaling pathway	KEGG:04668	0.057862959

Supplemental Table ST4. Individual integrated analysis of downregulated DEMs and upregulated gene targets. KEGG pathway enrichment analysis upregulated gene target lists for each individual downregulated DEM using G:profiler with g:SCS multiple testing correction. All significantly enriched (padj < 0.1) pathways are outlined in the table above.

Upregulated DEMs	KEGG Pathways Associated with Downregulated DEG Targets	KEGG ID	padj
miR-30b-5p	T cell receptor signaling pathway	KEGG:04660	0.000947305
	Natural killer cell mediated cytotoxicity	KEGG:04650	0.001402189
	Fc epsilon RI signaling pathway	KEGG:04664	0.026730659
	p53 signaling pathway	KEGG:04115	0.032777264
	Antigen processing and presentation	KEGG:04612	0.041433881
	Platelet activation	KEGG:04611	0.088744355
	Osteoclast differentiation	KEGG:04380	0.090220796
miR-29a-3p	Viral protein interaction with cytokine and cytokine receptor	KEGG:04061	0.001882935
	Cytokine-cytokine receptor interaction	KEGG:04060	0.002466077
	Cell adhesion molecules	KEGG:04514	0.01007563
	Intestinal immune network for IgA production	KEGG:04672	0.019628816
	Calcium signaling pathway	KEGG:04020	0.030962641
	Arrhythmogenic right ventricular cardiomyopathy	KEGG:05412	0.060698231
miR-3068-3p	none	none	none
miR-429-5p	Aldosterone synthesis and secretion	KEGG:04925	0.001981368
	Primary immunodeficiency	KEGG:05340	0.012794156
	Intestinal immune network for IgA production	KEGG:04672	0.017441974
	cAMP signaling pathway	KEGG:04024	0.017739638
	Calcium signaling pathway	KEGG:04020	0.02554256
	Endocrine and other factor-regulated calcium reabsorption	KEGG:04961	0.035590286
	Cytokine-cytokine receptor interaction	KEGG:04060	0.043905712
	Salivary secretion	KEGG:04970	0.071045759
	Longevity regulating pathway	KEGG:04211	0.076085793
miR-181d-5p	none	none	none
miR-181a-5p	cGMP-PKG signaling pathway	KEGG:04022	0.000391696
	Aldosterone synthesis and secretion	KEGG:04925	0.002966783
	Pancreatic secretion	KEGG:04972	0.004266939
	Oxytocin signaling pathway	KEGG:04921	0.009287143
	Rap1 signaling pathway	KEGG:04015	0.02673631
	Human T-cell leukemia virus 1 infection	KEGG:05166	0.036985653
	Gastric acid secretion	KEGG:04971	0.070900992
	Salivary secretion	KEGG:04970	0.093226813
	Longevity regulating pathway	KEGG:04211	0.099822794
miR-429-3p	Cholinergic synapse	KEGG:04725	0.012936289
	Platelet activation	KEGG:04611	0.015130886
	Tight junction	KEGG:04530	0.029317625
	Proteoglycans in cancer	KEGG:05205	0.040735923
	Ras signaling pathway	KEGG:04014	0.055388888
	Human T-cell leukemia virus 1 infection	KEGG:05166	0.058776085
	Calcium signaling pathway	KEGG:04020	0.05976168

	Prion disease	KEGG:05020	0.071662767
miR-26b-5p	Regulation of actin cytoskeleton	KEGG:04810	0.000580681
	MAPK signaling pathway	KEGG:04010	0.001709519
	Apelin signaling pathway	KEGG:04371	0.004596243
	Chemokine signaling pathway	KEGG:04062	0.012015271
	Proteoglycans in cancer	KEGG:05205	0.013790694
	Rap1 signaling pathway	KEGG:04015	0.017111431
	Pathways in cancer	KEGG:05200	0.018094193
	Ras signaling pathway	KEGG:04014	0.021727457
	Human T-cell leukemia virus 1 infection	KEGG:05166	0.023717917
	p53 signaling pathway	KEGG:04115	0.049984194
	Viral myocarditis	KEGG:05416	0.061597596
	Th1 and Th2 cell differentiation	KEGG:04658	0.072718341
	Longevity regulating pathway	KEGG:04211	0.074379604
	Fc gamma R-mediated phagocytosis	KEGG:04666	0.076058946
	Dilated cardiomyopathy	KEGG:05414	0.079471775
	Viral protein interaction with cytokine and cytokine receptor	KEGG:04061	0.081205219
miR-98-3p	Progesterone-mediated oocyte maturation	KEGG:04914	0.029060472
	Aldosterone synthesis and secretion	KEGG:04925	0.039220299
	Parathyroid hormone synthesis, secretion, and action	KEGG:04928	0.043207515
	Oocyte meiosis	KEGG:04114	0.050856889
	Cell cycle	KEGG:04110	0.057224058
	Apelin signaling pathway	KEGG:04371	0.071046384
	Oxytocin signaling pathway	KEGG:04921	0.084038985
miR-3535	Cell adhesion molecules	KEGG:04514	0.01235749
	NOD-like receptor signaling pathway	KEGG:04621	0.022549381
	Rap1 signaling pathway	KEGG:04015	0.02673631
	Regulation of actin cytoskeleton	KEGG:04810	0.029003959
	Pertussis	KEGG:05133	0.074741616
miR-381-3p	MAPK signaling pathway	KEGG:04010	0.010834651
	Progesterone-mediated oocyte maturation	KEGG:04914	0.029060472
	Parathyroid hormone synthesis, secretion, and action	KEGG:04928	0.043207515
	Oocyte meiosis	KEGG:04114	0.050856889
	Cell cycle	KEGG:04110	0.057224058
	Apelin signaling pathway	KEGG:04371	0.071046384
	Oxytocin signaling pathway	KEGG:04921	0.084038985

Supplemental Table ST5. Individual integrated analysis of upregulated DEMs and downregulated gene targets. KEGG pathway enrichment analysis downregulated gene target lists for each individual upregulated DEM using G:profiler with g:SCS multiple testing correction. All significantly enriched (padj < 0.1) pathways are outlined in the table above.
